# Flow-Induced Crystallization in Polyethylene: Effect of Flow Time on Development of Shish-Kebab

**DOI:** 10.3390/polym12112571

**Published:** 2020-11-02

**Authors:** Ruijun Zhao, Zhaozhe Chu, Zhe Ma

**Affiliations:** Tianjin Key Laboratory of Composite and Functional Materials, School of Materials Science and Engineering, Tianjin University, Tianjin 300350, China; rj_zhao@tju.edu.cn (R.Z.); chuzhaozhe@163.com (Z.C.)

**Keywords:** flow, crystallization, polyethylene, shish-kebab, relaxation

## Abstract

The flow-induced formation and relaxation of the representative oriented shish-kebab structure were studied with synchrotron small-angle X-ray scattering (SAXS) method. The flow duration was varied from 2 to 6 s at an identical strain rate to reveal the effect of flow time on stability and dimension of formed shish. It was found that the short flow time of 2 s was able to generate shish during flow, which, however, relaxed during the isothermal process after cessation of flow. An increase in flow time can improve the shish stability and the long flow time of 6 s can generate the stable shish that nucleate the growth of kebab lamellae. In addition, the quantitative analysis of SAXS results showed that with increasing flow time from 2 to 6 s, the shish length increased from 242 to 574 nm, while the shish diameter remained around 34 nm. This detailed information of the formed shish-kebab structure can be used to shed light on their evolution that occurred during flow from 2 to 6 s, where shish grew at a longitudinal speed of around 80 nm/s, and there was an improvement in the stability and nucleation capability for kebab lamellae.

## 1. Introduction

More than two-thirds of the polymeric materials fall into the category of crystallizable macromolecules. When these semi-crystalline polymers are processed from the melt state into the final product, the practical processing factors strongly influence the solidification rate, crystal modification, crystallite texture and orientation, etc. Among many processing parameters, flow, implementing shear or/and elongation deformation, are always employed in practice [1,2,3,4,5,6,7]. It is well recognized that flow can largely enhance crystallization rate by order of magnitudes and, more importantly, induce the formation of the special oriented crystallite structure, that is, shish-kebab, to improve the mechanical properties such as stiffness of the end-use products [8]. In this case, the corresponding flow-induced crystallization becomes a crucial subject in polymer physics and engineering, which determines the microstructure and macroscopic performance of materials.

The typical-oriented shish-kebab structure is composed of the fibrillar core of shish and the resulting lamellar crystals of kebab that are nucleated on the shish surface and grow radially along the direction perpendicular to the shish longitudinal axis [3]. For such a structure, the flow-generated shish determines the density of nucleation sites [9] and provides the orientation template for lamellar growth [10]. In reality, the formation of shish has been studied for more than half a century [11]. It has been well recognized that the external flow strength should overcome the intrinsic segmental relaxation, in order to be able to effectively stretch the polymer chain, as quantified by the classification of van Meerveld et al. [12] that the corresponding Weissenberg number based on the Rouse time ($\mathrm{Wi}=\dot{\gamma }\times \tau_{Rouse}$) should be larger than 1. Molecular stretch is the consequence of competition between the molecular mobility and external flow strength, so high molecular weight fraction with the relatively long relaxation time was often added to enhance the generation of shish.

There are many investigations reported to demonstrate the availability of this strategy [4,9,10,13,14,15,16,17]. The work of Seki et al. showed that the long chain-long chain entanglement played an important role in flow-induced crystallization and the number of generated threads increased with increasing the concentration of long chains in a nonlinear manner [15]. Cui et al. found that for the formation of shish nuclei, the critical strain decreased with the increase in the concentration of long chains [4]. Although it was thought in the early studies that it is the high molecular weight fraction added that aggregate to form shish, the milestone work of small-angle neutron scattering on the designed isotactic polypropylene with different deuterium-labeling chain systems performed by Kimata et al. [18] demonstrated that shish had the same fraction of high molecular weight as the bulk melt. It is likely that those polymer chains with the high molecular weight are easy to be deformed by flow, because of the relative long relaxation time, and their main role in the flow-induced formation of shish is to recruit all chains in the melt to aggregate to form the ultimate shish [15].

Next, the detailed formation mechanism was focused on to achieve the control of the flow-induced structure. Combing the small and wide-angle X-ray scattering with the rheological response, Cui et al. [19] investigated the structural formation at multi-length scales. In that work, the ordering pathway proposed includes several steps in different length scales, following the coil in melt → stiff segment of helix → precursor → crystal. On the other hand, it was also proposed that the formation of shish went through multi-steps. Hashimoto et al. [20] found the first occurrence of liquid–liquid phase separation generated some domains with concentrated chains and the stretched chains connecting neighboring domains grew into these domains to form shish. Mykhaylyk et al. [21] proposed that flow first induced the generation of some point-like nuclei and they further aligned to form shish. Differently, it was proposed that shish needed the necessary step of growth to develop into the ultimate fibrillar shish, though the original structures could be different, for example, the flow-induced nuclei or point-like precursors [22,23,24]. Recently, it was unexpectedly found that when flow stopped, the formed shish may not nucleate the growth of kebab, but relax it [25]. This implies that the shish that are generated during flow are not always stable after flow, where the polymer chain may quickly relax back to the random coil conformation. Then, the stability of shish influences their nucleation capability of the oriented kebab lamellae. Actually, the shish stability cannot be directly characterized by the in situ method applied to monitor the structure evolution during flow. In this case, the isothermal process after flow must be tracked to examine shish stability. Balzano et al. employed the small-angle X-ray scattering to monitor the dissolution kinetics of shish in a bimodal polyethylene system and found that the relaxation behavior at the high temperature of 142 °C even over the equilibrium melting temperature of polyethylene, was closely associated with the reptation process of the high molecular weight chains [26]. This suggests that the stretched high molecular weight polyethylene chains form the backbones of shish. Furthermore, the quantified activation energy of the relaxation process can provide more detailed information on the relaxation. It was reported for some flow-induced structures that the activation energy of ordering relaxation is much larger than that of chain mobility, indicating that relaxation of flow-induced structure also involves other factors, such as the helical interactions [27,28]. So far, concerning the representative shish-kebab structure, the stability of shish is not clear enough.

In this work, the post-flow evolution of flow-generated shish was investigated with the time-resolved small-angle X-ray scattering. Three flow times of 2, 4, and 6 s were employed at the identical strain rate, of which the results obtained immediately after flow could reflect the formation during flow, and the results obtained during the subsequent isothermal process could give the information on the stability of the formed shish. It was found that although the applied duration of 2–6 s was able to generate shish, their stability and dimension exhibited a strong dependence on the flow time. The evolution of stability and dimension indicated that during flow, the unstable shish were generated and further developed to improve the stability with growth along the longitudinal direction.

## 2. Experimental Section

### 2.1. Material

The material studied in this work was a bimodal polyethylene sample that was prepared by the solution blending of ultrahigh molecular weight polyethylene (UHMWPE, *M*_w_ = 1480 kg/mol, *M*_w_/*M*_n_ = 2) [29] and the low molecular weight polyethylene (LMWPE, *M*_w_ = 45 kg/mol, *M*_w_/*M*_n_ = 3) matrix. The weight fraction of UHMWPE in the bimodal system was 5%. In order to reach mixing at the molecular level, UHMWPE and LMWPE raw materials were dissolved into the hot xylene. After being stirred in nitrogen for 1 h, the solution was poured into cold methanol. The precipitate was filtered and washed with methanol. Then, the mixture antioxidants of Irganox 1010 and Irgafos 168 (2000 ppm) were added into the blend. Finally, the precipitate was dried in the vacuum condition at 80 °C for 2 days. For such a bimodal blend, the critical overlapping concentration (c*) of UHMWPE chains can be estimated by c* = 3*M*_w_/(4π[〈*R*_g_^2^〉^1/2^]^3^*N*_a_) [30], where 〈*R*_g_^2^〉 is the mean-square radius of gyration and *N*_a_ is Avogadro’s number. According to 〈*R*_g_^2^〉^1/2^/*M*_w_^1/2^ = 0.46 Å/[(g/mol)^1/2^] [31], the critical overlap concentration c* = 0.33 wt% for the long-chain in this work. In this case, the high UHMWPE concentration of 5 wt % could ensure that the UHMWPE long chains could build entanglements not only with the low molecular weight matrix but also with long chains themselves. On the other hand, the presence of a low molecular weight matrix could participate in the formation of crystallites and also facilitate the shear deformation of material.

### 2.2. Method

The shear flow was implemented by a customized shear cell with the rotational plate–plate geometry, where the Kapton windows were employed to allow the X-ray scattering signal collected by the detector. Although the rotational plate had a radius of 15 mm, the X-ray window was located 8 mm away from the plate center. The sample thickness was around 0.5 mm. Time-resolved small-angle X-ray scattering (SAXS) measurements were carried out at beamline BL16B of the Shanghai Synchrotron Radiation Facility (SSRF). The X-ray wavelength was 1.033 Å and the beam size was approximately 300 μm × 400 μm. SAXS measurements were performed with a detector (512 × 512 pixels with a pixel size of 260 μm × 260 μm) placed at a distance of 7432 mm. The acquisition period was 10 s for each SAXS pattern. The 2D scattering images obtained were processed with Fit2D software [32]. The resolution of the scattering vector *q* was around 0.002 nm^−1^.

**Figure 1:**
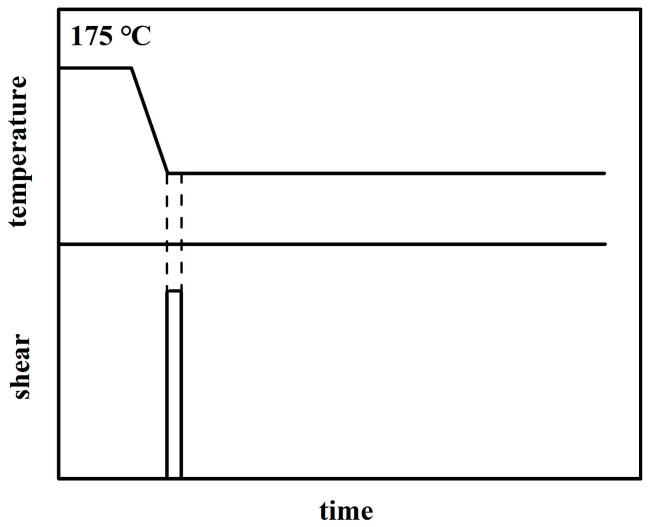
Scheme of the experimental protocol.

Figure 1 shows the experimental protocol. The polymer was first held at 175 °C much higher than the equilibrium melting temperature of polyethylene for 10 min, in order to erase thermal and processing histories. Then, the relaxed melt was cooled at −10 °C/min to the isothermal temperature of 133 °C. After 133 °C was stabilized, a shear flow was imposed at the shear rate of 120 s^−1^, where the flow time *t*_s_ was varied from 2 to 4 and 6 s. After flow, the subsequent isothermal crystallization was monitored by the in situ SAXS method.

## 3. Results and Discussion

**Figure 2:**
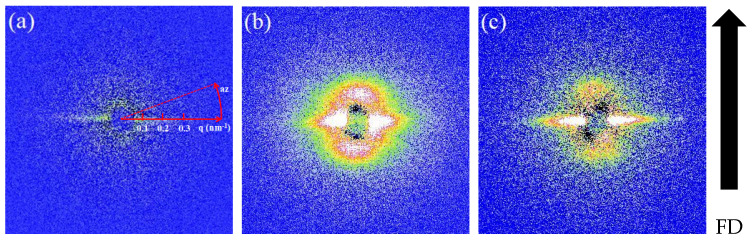
The 2D small-angle X-ray scattering (SAXS) patterns obtained immediately after flow with different durations of (**a**) 2, (**b**) 4, and (**c**) 6 s. The flow direction (FD) is vertical.

Figure 2 shows the 2D SAXS patterns obtained immediately after the shear flows, which have distinct durations from 2 to 4 and 6 s at an identical shear rate of 120 s^−1^. It could be clearly seen that the characteristic SAXS streaks appeared in the equatorial region of all 2D SAXS patterns obtained. This demonstrates that the fibrillar shish, which are composed of stretched chains oriented along the flow direction and possess a sufficiently higher density than the surrounding melt, are formed by the applied flows. In addition, it is also noted that for the relatively longer flow durations of 4 and 6 s, the tear-drop shaped scattering signals are observed in the meridional region (Figure 2b,c). These characteristic meridional scattering signals originated from the periodically alternated kebab lamellae with the intermediate amorphous region. Based on the SAXS results, it is disclosed that with 120 s^−1^ employed in this work, a flow of 2 s is strong enough to stretch polymer chains to generate shish and the subsequent elongation of flow to 4 and 6 s can further nucleate the growth of kebab lamellae on the shish surface.

**Figure 3:**
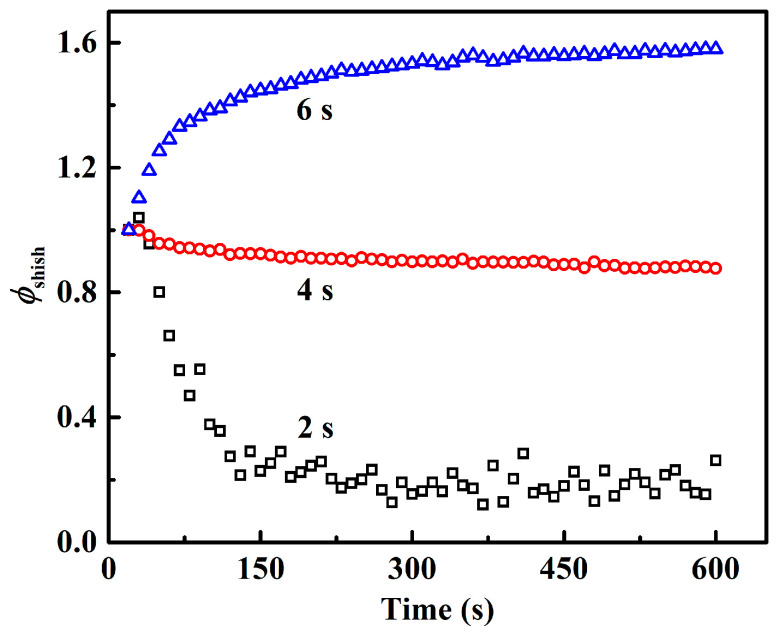
Time evolution of the relative shish integrated intensity under different shear conditions.

The above results show that the flows of 2–6 s are able to induce the formation of shish during flow, but more detailed properties of these formed shish, for example, whether they relax or continuously grow after flow, cannot be revealed. To examine the stability of flow-generated structures, the evolution of shish is investigated for the isothermal process after flow. To quantify the evolution of characteristic SAXS signals, the intensities of SAXS streaks are integrated over equatorial region *I*_eq_ = $\int_{0.018}^{0.5}\int_{-15{}^{\circ}}^{15{}^{\circ}}I\left(\mathrm{az},q\right)$*d*_az_*d*_q_, with *az* being the azimuthal angle and *q* being the alternate scattering vector. The alternate scattering vector *q* = 4πsinθ/*λ*, 2*θ* the scattering angle and *λ* the X-ray wavelength. In order to more clearly present the evolution tendency of shish, the integrated SAXS intensities of equatorial streaks are normalized by their initial values at the beginning of the isothermal process after flow as follows:

(1) $$\phi_{\mathrm{shish}}=\frac{I_{\mathrm{eq}}(t)}{I_{\mathrm{eq}}(0)}$$

where *I*_eq_(0) and *I*_eq_(t) are the integrated intensities of the equatorial streaks at the moment when the flow stops and at the isothermal time *t*, respectively. Figure 3 shows the change of shish integral intensities over the isothermal time. It is clear that the post-flow densities of the flow-generated shish are strongly dependent on flow time. For flow time of 2 and 4 s, the equatorial streak intensity decays within the isothermal process after flow, demonstrating that these formed shish are unstable. It should also be noted that the shish generated by 2-s flow relaxed almost completely, while shish formed with 4-s flow decreased by a very small percentage. It is indicated that during the flow period from 2 to 4 s, the shish develop to improve the stability, although they do not become completely stable yet at the end of 4 s. In contrast, when flow time is prolonged to 6 s, the formed shish are stable and can continue to grow within the isothermal process after flow. For these flow-induced shish, their distinct properties are determined by the degree of evolution during flow. It has been revealed that the shish pathway may go through steps considering the ordering at different length scales including molecular stretch, conformation helix ordering, and local aggregation of segmental bundles [19,33]. The results of this work provide the information on shish development, where formed shish also need to evolve during flow to improve the stability.

**Figure 4:**
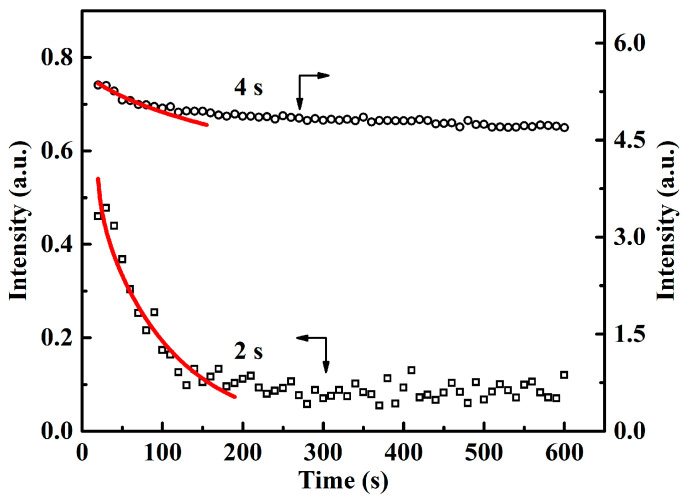
Time evolution of the equatorial streak integral intensity for flow time t_s_ of 2 and 4 s. The open symbols are experimental data and the red solid lines are the fitting results with the modified Doi–Edwards memory function.

For the unstable structure, their relaxation kinetics can further shed light on the degree of aggregation ordering. The decrease in equatorial intensity of *I*_eq_(t) are analyzed with the modified Doi–Edwards memory function [26]:

(2) $$I_{\mathrm{eq}}(t)=S_{y}\sum_{\mathrm{podd}}\frac{1}{p^{2}}\cdot \exp(-p^{2}\frac{t-t_{0}}{\tau_{D}})$$

where S_y_ is the vertical translation parameter, *t*_0_ is the reference time for shish to start the relaxation process, and *τ*_D_ is the characteristic relaxation time. As shown by Figure 4, the relaxation kinetics of shish generated by the flow of 2 and 4 s can be described by this memory function. However, the obtained characteristic relaxation times *τ*_D_ are different, which are 93 and 2886 s for the flow time of 2 and 4 s, respectively. This means that the shish generated by flows of 2 and 4 s follow different relaxation kinetics, which in turn indicates that these shish have different stability associated with the ordering degree. On the other hand, it is reported that for unstable shish, the stretched chains with the relatively higher molecular weights play a very important role in the formation and relaxation of shish, which act as the structural skeleton of shish. Then, the Rouse time *τ*_Rouse_ and reptation time *τ*_rep_ of the UHMWPE studied in this work can be estimated from the following formula:

(3) $$\tau_{\mathrm{Rouse}}=\tau_{e}Z^{2}$$

(4) $$\tau_{\mathrm{rep}}=3\tau_{e}Z^{3}{\left(1-\frac{1.51}{\sqrt{Z}}\right)}^{2}$$

where *τ*_e_ is the entanglement equilibration time, for PE *τ*_e_ = 7 × 10^−9^ s at 190 °C [34]. *Z* is the number of entanglements per chain (*Z* = *M*_w_/*M*_e_), where *M*_e_ is the molecular weight between the two adjacent entanglement points (*M*_e_ = 828 g/mol) [34]. Knowing the activation energy *E*_a_ = 21.8 kJ/mol [35], the reptation time and Rouse time of UHMWPE can be estimated for 133 °C applied in this work, which are *τ*_rep_ = 247 and *τ*_Rouse_ = 0.048 s, respectively. According to the rheological classification proposed by van Meerveld et al. [12], whether the molecular chain can be stretched depends on the Weissenberg number, *Wi*_s_ (=$\dot{\gamma }$τ_Rouse_), where $\dot{\gamma }$ is the shear rate. In this work, *Wi*_s_ is around 6, larger than 1, the strength of flow field is large enough to stretch the molecular chain, which is consistent with the observation of the highly oriented shish in the SAXS patterns. Herein, it is also found that for the unstable shish generated by flow of 2 s, the obtained characteristic relaxation time *τ*_D_ = 93 s is smaller than the intrinsic reptation time *τ*_rep_, that is, 247 s of the UHMWPE chains. More interestingly, for the flow of 4 s, the unstable shish relax partially and the corresponding characteristic relaxation time obtained by the fitting of memory effect raise significantly to 2866 s, much longer than that of UHMWPE [27,36]. It is known that for polymer chains, it takes the reptation time to move out of the original tube built by the constraint entanglements and in this way, to completely erase the initial structural features. It seems that for flow time of 2 s, the relaxation of generated shish may be associated with just part of the polymer chain. This is consistent with the previous investigations that for shish formation, it is not necessary to need the coil–stretch transition of the whole chain and stretching part of the chain can meet the requirement [4,37]. More importantly, the distinct difference in the characteristic relaxation time fitted indicates that the relaxation process of *t*_s_ = 4 s was more complex than that of *t*_s_ = 2 s. As flow time increases to 4 s, the shish relaxation involves not only polymer movement which was determined by the intrinsic rheological properties, but also the aggregation state (like bundles) of segments, which compactly and ordered packed to lower the free energy of the system to stabilize the flow-induced structures [28,36]. This is consistent with the following observation that when flow time was continuously increased to 6 s, the shish generated are stable. These results suggest that although the flow of 2 s is able to generate shish, the formed shish further improve the stability with the subsequent flow.

**Figure 5:**
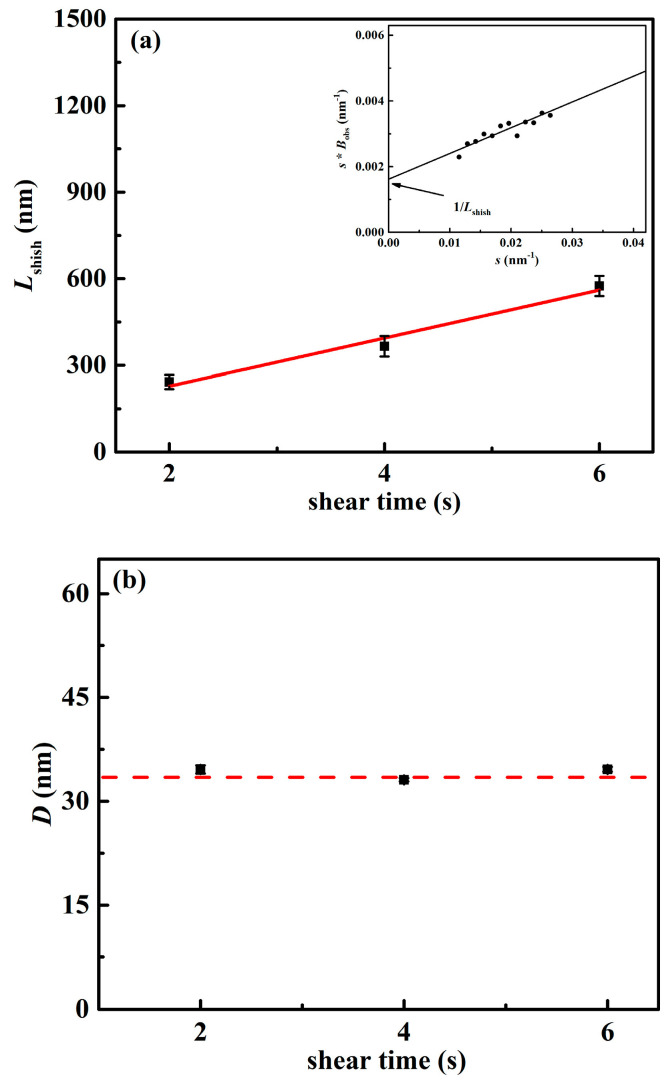
The change of shish (**a**) length *L*_shish_ and (**b**) diameter *D* with the shear time. The length and diameter of shish were obtained after the shearing stopped immediately. The red solid line is the fitting result. The red dashed line is used to guide the eye. The insets show examples of using Equation (5) to obtain the shish length *L*_shish_.

In addition to stability, the specific dimension of shish may also evolve during flow. For these formed shish, their length (*L*) and diameter (*D*) can be obtained by the analysis of SAXS results [9,38]. According to the SAXS deconvolution method proposed by Ruland [39], the azimuthal breadth *B*_obs_ of equatorial streaks is contributed by two components, including the shish length (*L*_shish_) and orientation (*B*_ϕ_), following the below quantitative relation:

(5) $$B_{\mathrm{obs}}=\frac{1}{L_{\mathrm{shish}}\times s}+B_{\phi }$$

where *B*_obs_ is the azimuth width at the scattering vector *s* (*s* = 2 sinθ/*λ*) along the equator. After multiplying both sides of Equation (5) by *s*, the shish length *L*_shish_ can be obtained from the reciprocal of the intercept of the fitting line *B*_obs_ × *s* vs. *s* (as indicated by Figure 5a inset). The results of Figure 5a clearly present that there are distinct differences in the length of formed shish. As flow duration is raised from 2 to 6 s, the shish length increases from 242 to 574 nm. Interestingly, the shish length seems to follow a linear dependence on the flow time. It is indicated that during the application of flow (2–6 s), shish grow along the longitudinal direction, which follows the growth rate of ~80 nm/s.

On other hand, the radius *R* of shish was determined using Guinier law [38,40,41]. In equatorial streak scattering, the relationship between scattering intensity *I*(*q*) at *q* values and alternate scattering vector *q* is:

(6) $$I(q)\propto \frac{2\pi }{q}\cdot \exp(-R_{C}^{2}\cdot q^{2}/2)$$

where *R*_c_ is the gyration radius of the cross-section (*R*_c_ = *R*/$\sqrt{2}$). *R*_c_ can be obtained by linear fitting of low *q* value region in ln[*I*(*q*)*q] vs. *q*^2^ graph. From Figure 5b, it is interesting to find that the shish diameter does not change with the increase in shear time and remains at around 34 nm, different from the aforementioned obvious increase in length. Phillips et al. studied the dimension evolution of shish in the nucleating agents filled system of isotactic polypropylene and also found the shish diameter reached the plateau size after an initial increase [38]. Based on these distinct changes of shish length and diameter, it is likely that the further elongation of flow duration from 2 to 6 s mainly affects the increase in shish length. In other words, the formed shish mainly develop along their longitudinal direction with a similar diameter within the flow lasting from 2 to 6 s.

From the above analysis, it could be seen that the flow time varying from 2 to 6 s exhibited an important impact on the stability and length of shish. Herein, it should be pointed out that these specific properties are likely to also depend on the composition of the bimodal blend. The investigation of Cui et al. showed that the critical strain of forming shish decreased with the increase in the concentration of long chains [4]. In addition, Keum et al. found that increasing the concentration of long chains could improve the stability of flow-induced shish [9]. Thus, the properties of flow-induced shish could be varied with the change of material composition.

**Figure 6:**
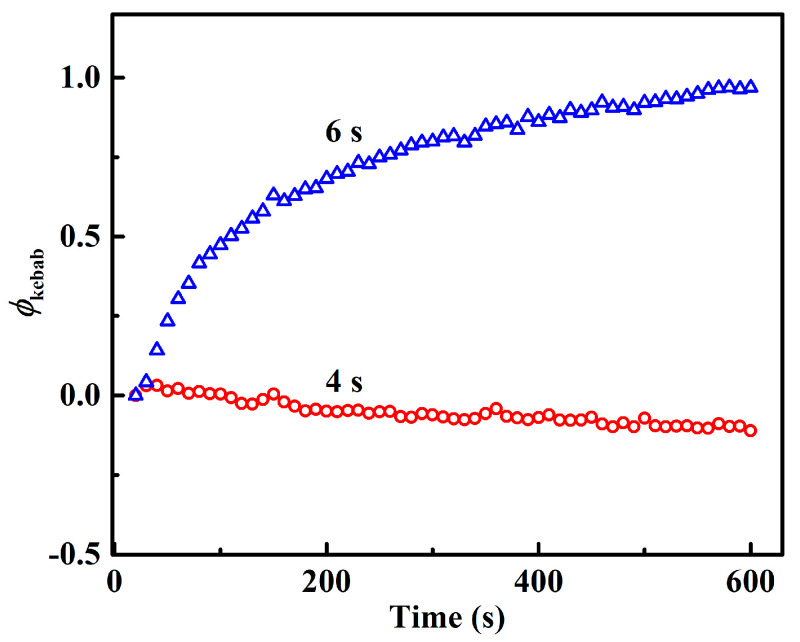
The evolution of integral meridional SAXS intensity during the isothermal process after flow with the flow time of *t*_s_ = 4 and 6 s.

Moreover, the kebab structure is also formed under different shearing conditions. To trace the evolution of kebab during the isothermal period after flow, the scattering intensity in the meridian direction of different 2D SAXS patterns are integrated. The expression of the meridian integral intensity *I*_me_ is *I*_me_ = $\int_{0.018}^{0.5}\int_{50{}^{\circ}}^{130{}^{\circ}}I\left(\mathrm{az},q\right)$*d*_az_*d*_q_. Next, the variation of integrated kebab intensity can be expressed by the following method $\phi_{\mathrm{kebab}}=\frac{I_{\mathrm{me}}(t)-I_{\mathrm{me}}(0)}{I_{\mathrm{me}}(0)}$, where *I*_me_(0) and *I*_me_(*t*) are the integrated intensity of the meridian streak at the moment when the flow stops and at time *t*, respectively. Figure 6 displays the evolutions of *ϕ*_kebab_ during the isothermal process after flow, where no meridional intensity of kebab is observed for 2-s flow. It is found that the evolutionary trend of kebab is similar to that of the corresponding shish. For a shear time of 2 s, there is no kebab signal observed during the whole isothermal period, even when shish do not relax. This indicates that although shish are generated by the flow of 2 s, they are not able to nucleate the growth of kebab during the subsequent isothermal process. When *t*_s_ = 4 s, kebab lamellae are generated with the flow, but the integral intensity shows a slow decrease after flow, which is essentially similar to the decrease in corresponding shish structure (*t*_s_ = 4 s). During flow, polymer chains are oriented and stretched, the kebab also grows. However, after flow, polymer segments can quickly relax, as indicated by the very short relaxation time of only 0.048 s. However, when the shear time is 6 s, kebab integral intensity increases rapidly at first, and then increases slowly. The stability of both shish and kebab have the similar thermal dependence (to melt or grow simultaneously) on flow time.

**Figure 7:**
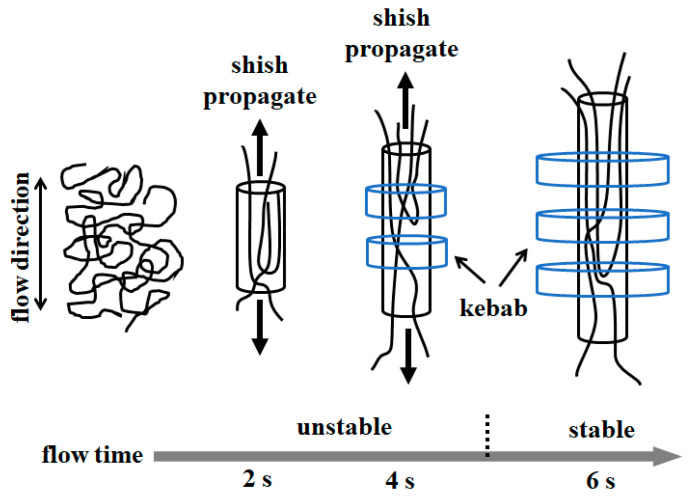
Schematic illustration of the development of shish-kebab structure affected by different flow time.

Based on the above results of the properties of shish and kebab with different shear durations, it can be seen that the formation of the shish-kebab structure is completed in multiple steps. Now, we can consider these three independent experiments as a continuous shearing and obtain the shish-kebab formation process as follows (see Figure 7). When the flow time is short (0 < *t*_s_ < 2 s), long-chain molecules are easier to be deformed because of the relatively long relaxation time for the identical flow field controlled by the macroscopic shear rate. The long chains at sufficient concentration beyond the critical overlapping concentration, mainly form the skeleton of shish, and recruit other short chains to aggregate to form the ultimate shish. During this first period, the length of shish reaches 242 nm with a diameter of around 34 nm, while they are not able to nucleate the growth of kebab lamellae. As shear continues (2 s < *t*_s_ < 4 s), shish grow further in a longitudinal direction to about 365 nm with a constant diameter of 34 nm. In the meantime, shish nucleate the appearance of kebab lamellae, which grow along a perpendicular direction with respect to that of shish. As flow is continuously applied to 6 s, shish have more time to grow and reach 574 nm at the end of the flow. Compared with the aforementioned shish generated by 4-s flow, these shish provide more surface to nucleate the growth of kebab lamellae.

## 4. Conclusions

We examined the stabilities and dimensions of the flow-induced shish-kebab structure by monitoring the isothermal process after flow by the synchrotron small-angle X-ray scattering. The results obtained just after flow demonstrated that flow time of 2 s at a strain rate of 120 s^−1^ can generate the fibrillar shish composed of the stretched segments. However, these flow-induced shish were unstable and relaxed during the isothermal process. As the flow time was increased, shish formed had higher stability and became stable for flow time of 6 s. Moreover, results of SAXS quantitative analysis showed that the formed shish had the same diameter but different lengths, indicating an average speed of around 80 nm/s for shish longitudinal growth during flow. In summary, the increase in flow time from 2 to 6 s not only improves the shish stability and nucleation capability for kebab lamellae, but also increases the longitudinal dimension of shish.
